# Viruses That Can and Cannot Coexist With Humans and the Future of SARS-CoV-2

**DOI:** 10.3389/fmicb.2020.583252

**Published:** 2020-09-18

**Authors:** Yuki Furuse, Hitoshi Oshitani

**Affiliations:** ^1^Institute for Frontier Life and Medical Sciences, Kyoto University, Kyoto, Japan; ^2^Hakubi Center for Advanced Research, Kyoto University, Kyoto, Japan; ^3^Department of Virology, Tohoku University Graduate School of Medicine, Sendai, Japan

**Keywords:** COVID-19, SARS-CoV-2, epidemiology, transmission, zoonosis, evolution

## Abstract

Severe acute respiratory syndrome coronavirus 2 (SARS-CoV-2) has become a worldwide pandemic. Many projections concerning the outbreak, such as the estimated number of cases and deaths in upcoming months, have been made available. However, what happens to the virus after the pandemic subsides has not been fully explored. In this article, we discuss the ways that past and present human viruses have emerged via zoonotic transmission, the mechanisms that they have acquired the ability for effective transmission among humans, the process to sustain a chain of transmission to coexist with humans, and the factors important for complete containment leading to eradication of viruses. These aspects of viral disease may provide clues for the future path that SARS-CoV-2 might take in relation to human infection.

## Introduction

**FIGURE 1:**
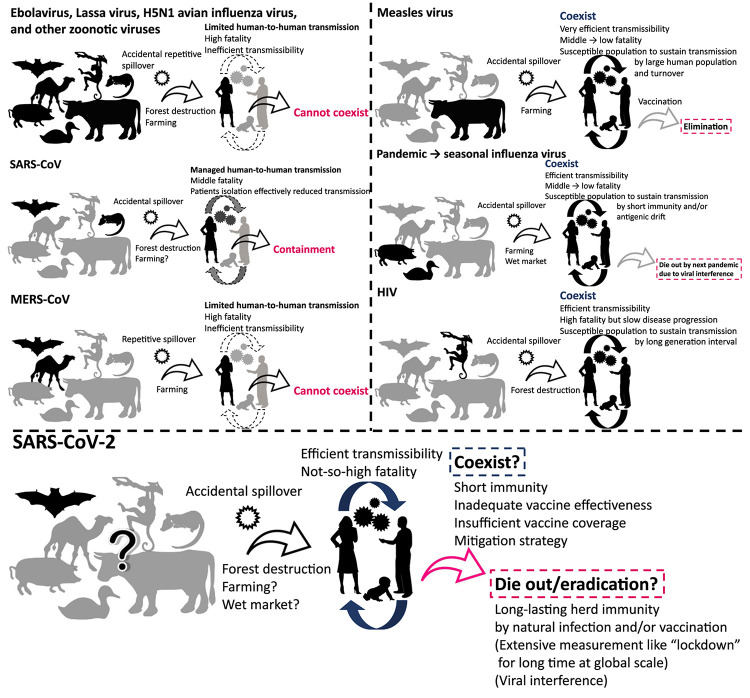
Viruses that cannot coexist and that have coexisted with humans. The figure illustrates schematic overview of the history of emergence, adaptation, transmission, and consequence of viruses.

The emergence of severe acute respiratory syndrome coronavirus 2 (SARS-CoV-2) was reported from China in December 2019. As of August 2020, the world is gripped by a pandemic of the virus, with numbers of cases and deaths increasing globally. People are trying to reduce new infections by non-pharmaceutical intervention (Flaxman et al., 2020; Hsiang et al., 2020; Lai et al., 2020). A global research effort is underway for the development of specific remedies and vaccines for the virus (European Centre for Disease Prevention and Control, 2020; Wiersinga et al., 2020). Many projections concerning the outbreak, such as the estimated number of cases and deaths in upcoming months, have been made available (Holmdahl and Buckee, 2020; Jewell et al., 2020; Metcalf et al., 2020). However, what happens to the virus after the pandemic subsides has not been fully explored. What can we expect in the future, and will the virus coexist with us or go extinct? (Figure 1).

SARS-CoV-2 is believed to have been introduced by zoonotic transmission (Lu et al., 2020; Wu et al., 2020; Zhou et al., 2020). Zoonosis, which is identified as a disease transmitted from animals to humans, is a major route of introduction of new viruses to humans. Zoonotic viruses were repeatedly introduced to people as a spillover from natural hosts. However, many of them cannot sustain a chain of transmission in human populations for extended periods. On the other hand, some viruses, such as pandemic influenza virus and human immunodeficiency virus (HIV), have adapted to humans after zoonotic introduction, acquiring the ability to sustain human-to-human transmissions (Wasik et al., 2019). In this article, we provide an overview and discuss how past and present human viruses emerged, spread, and persisted or disappeared after zoonotic introduction. These aspects of viral disease may provide clues for the future path that SARS-CoV-2 might take in relation to human infection.

## Zoonotic Transmission, Adaptation to Humans, and Eradication of Viruses

The chance of zoonotic transmission of virus increases by frequent contact between humans and animals. This phenomenon could be partially attributed to forest destruction for urban development and exploitation of natural resources (Figure 1; Kessler et al., 2018; Gibb et al., 2020). The emergence of HIV and repetitive spillover of Ebolavirus might be caused by such activities (Whiteside and Zebryk, 2015). The transmission of simian immunodeficiency virus to humans and its subsequent adaptation led to the generation of HIV (Sharp and Hahn, 2011). Farming is an obvious occupation where human-animal contact is likely to occur on a regular basis. Zoonotic transmission of animal viruses from farm animals to humans may have resulted in the emergence of pandemic influenza and measles viruses (Webster et al., 1992; Düx et al., 2020). In the past ∼100 years, we witnessed the emergence of pandemic influenza caused by introductions of novel viruses from avian or swine species (Webster et al., 1992): Spanish flu in 1918, Asian flu in 1957, Hong Kong flu in 1968, and “Swine flu (H1N1pdm09 virus)” in 2009. The descendant viruses of Hong Kong flu and Swine flu now coexist in human communities as seasonal influenza (Morens and Taubenberger, 2011; Taubenberger et al., 2019). Measles virus was introduced by (relatively) ancient zoonotic transmission that has acquired its exclusive circulation in humans; phylogenetic analyses suggest that a bovine virus was transmitted to humans 1,000–2,500 years ago, leading to its adaptation as measles virus (Furuse et al., 2010; Düx et al., 2020). In addition, wet market selling of live animals is often recognized as the source of zoonotic virus transmission to humans (Cowling et al., 2013). Modern-day extensive forest destruction and large-scale farming likely increase the risk of zoonotic transmission of novel viruses to humans (Dobson et al., 2020; Gibb et al., 2020). Further, urbanization and globalization of today’s world make outbreaks that could have been contained in a local area easily and rapidly spared to other parts of world (Neiderud, 2015; Wu et al., 2017). One health approach including surveillance system to detect outbreaks of unknown disease in humans and animals at a global level must be important for better preparedness in future (Watsa, 2020).

Establishing infection in new hosts often requires numerous adaptive changes, such as receptor-specificity adjustments (Li et al., 2005; Yamada et al., 2006; Taubenberger and Kash, 2010), optimizing the compatibility with host’s factors (Long et al., 2016), and overcoming host antiviral defenses (Sawyer et al., 2004; Rajsbaum et al., 2012; Sauter and Kirchhoff, 2019). For example, when avian influenza virus adapted to humans, mutations in the HA gene altered avidity for cellular receptor from avian-type to human-type, mutations in the PB2 gene increased viral polymerase activity in human cells by interaction with a host’s factor, ANP32A, and mutations in the NS1 gene regulated innate immunity in a species-specific manner (Long et al., 2019). Detailed virological and physiological mechanisms of genetic mutations that affect adaptation to the new hosts are reviewed in Wasik et al. (2019); Letko et al. (2020). Also, we explore another aspect of farming that might contribute to zoonotic transmission. During viral replication in host cells, genetic mutations are randomly induced into viral genomes (Bordería et al., 2011). Yet, generated genetic diversity cannot be effectively transmitted from infector to infectee due to the so-called bottleneck effect (Domingo et al., 1996). However, when farm animals are raised in densely crowded conditions, an inter-host genetic bottleneck may be loosened (Domingo and Holland, 1997). Virus with mutations that enhance infectivity in humans may be induced and transmitted under such conditions, increasing the chances that a mutant virus will establish infection in a person.

**FIGURE 2:**
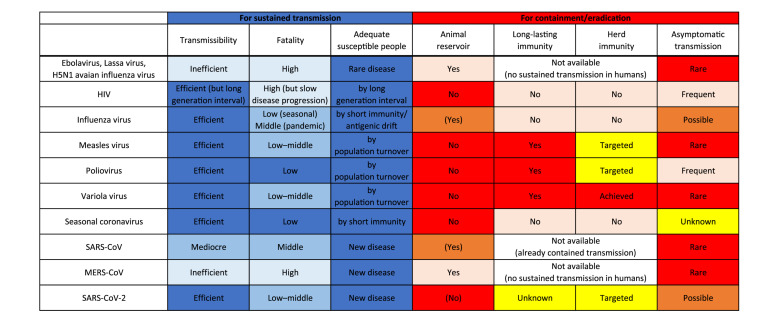
Factors responsible for sustained transmission and containment/eradication of viruses. Deeper blue colors indicate more adapted characteristics for sustained transmission in humans, and deeper red colors indicate more favorable features for containment/eradication of viruses.

After introduction to humans, viruses must sustain a chain of transmission to coexist as “human viruses” (Figures 1, 2). First, transmission should be efficient enough that the reproduction number, the average number of secondary cases per infectious case, is >1 (Delamater et al., 2019). This ability might be closely tied to achieving efficient replication ability in new hosts via genetic mutations. Second, viruses should not consume susceptible hosts; a certain fraction of susceptible hosts is needed to sustain transmission. Measles virus is known for great transmission efficiency due to air-borne transmission (Guerra et al., 2017; National Center for Immunization and Respiratory Diseases, 2017). When a virus is highly transmissible, shows a short generation interval, and induces life-long immunity; size of host population and turnover are important for sustaining viral transmission. Mathematical models show that a population of 250,000–500,000 is needed to maintain viral transmission (Black, 1966). For influenza virus, short-lived acquired immunity and viral evolution called antigenic drift are considered to allow escape from host immunity (Ferguson et al., 2003; Smith et al., 2004) although the duration of protective immunity against the virus is controversial (Couch and Kasel, 1983; Xia et al., 2005; Kucharski et al., 2015). These properties encourage sustained transmissions by creating an adequate proportion of susceptible hosts. Long generation interval due to chronic infection is considered another way that viruses maintain a population of susceptible hosts, as exemplified by HIV (Gran et al., 2008). The virus is known for its long latent phase followed by slow disease progression to acquired immunodeficiency syndrome, which takes more than a decade. Third, viruses should not kill too many infected individuals to maintain an ecologically fit life cycle. Notably, mortality of newly introduced viruses can decrease after a certain period of circulation as the pandemic influenza transforms into a seasonal influenza, possibly due to immune response to reinfection and viral adaptation in humans in post-pandemic epidemics (Barry et al., 2008; Dawood et al., 2012; Lin et al., 2012). Similarly, mortalities of measles and smallpox are reportedly high in naïve populations, but less in populations where such viruses coexist (Elwood, 1989; Shanks et al., 2011).

In contrast, zoonotic viruses, such as Ebolavirus, Lassa virus, and H5N1 avian influenza virus, have not adapted well enough to humans to achieve effective transmission. Host susceptibility is inadequate, viral replication ability is inefficient, and/or fatality is too high. Case fatality rates of these viral zoonoses in the accidental host, humans, were extraordinarily high, ranging over 10–90% (Heeney, 2006). Such severe illness reduces the chance of transmission by death, hospitalization, and decreased mobility of patients. Inefficient transmission can also be attributed to the mode of transmission such as blood- (and other body fluids-) borne transmission for some viruses including Ebolavirus (Chowell and Nishiura, 2014). These factors result in failure to establish a sustained chain of transmission (Figures 1, 2).

Viruses that once established sustained human-to-human transmission can disappear. A good example is a replacement of previously circulating seasonal influenza virus by newly emerged pandemic influenza virus. When a human-adapted influenza virus emerged after zoonotic transmission of avian or swine influenza virus as a pandemic virus, the seasonal human influenza virus that had been circulating before will disappear. Then, the pandemic virus becomes the “new” seasonal human influenza virus (Webster et al., 1992). This phenomenon is likely due to interference between the viruses (Palese and Wang, 2011; Furuse and Oshitani, 2016). Vaccination also can eradicate viruses. Smallpox variola virus has been identified as the first and currently the only human virus eradicated by vaccination. Meanwhile, measles virus and poliovirus remain to be the target for eradication by vaccination. Several important factors are required for successful eradication: (1) virus should circulate only among humans; that is, no animal reservoir can exist; (2) vaccine should produce a long-lasting immunity; (3) vaccination coverage should be high enough to achieve herd immunity at a global scale; and (4) most infections should cause symptomatic illness. Variola virus was able to meet all the four conditions, but unmet factors (3) and (4) for poliovirus and unmet factor (3) for measles virus make the ongoing effort to eradicate them challenging (Figure 2; Kew et al., 2005; Moss and Strebel, 2011; Furuse and Oshitani, 2017; Chard et al., 2020).

## Coronaviruses Including SARS-CoV-2

Seasonal human coronaviruses, including 229E, OC43, NL63, and HKU1, can cause common cold-like respiratory symptoms, circulating exclusively in humans. Members of the family Coronaviridae, however, can be found in both humans and other animals (Vijaykrishna et al., 2007). Hence, an ancient zoonotic origin of seasonal human coronaviruses is likely (Forni et al., 2017). Severe acute respiratory syndrome coronavirus (SARS-CoV) caused a multi-country outbreak in 2003. Its closely related virus was found in bats, and civets were considered as an intermediate host for transmission of the virus from bats to humans (Shi and Hu, 2008). Viral infection was sustained by sequential human-to-human transmissions (Anderson et al., 2004). The virus is often found to cause severe illness, and transmissibility becomes high at the time when patients develop severe symptoms (Anderson et al., 2004). Thanks to these characteristics, sensitive detection and timely isolation of patients, along with appropriate standard precautions in nosocomial settings, can effectively reduce new transmission (Fraser et al., 2004). As a result, the outbreak was contained within a year (Figures 1, 2). Middle East respiratory syndrome coronavirus (MERS-CoV) was introduced by zoonotic transmission from camels; the camel virus was reported to be likely originated from a bat virus (Mohd et al., 2016). Whereas some instances of human-to-human transmission of the virus did occur within households and in nosocomial settings, overall transmission was not sufficient to sustain transmission in humans (Figures 1, 2; Raj et al., 2014). Still, the high prevalence of the virus in domesticated camels caused repetitive spillover of the virus (Azhar et al., 2014).

SARS-CoV-2 is also believed to have come from a bat virus (Boni et al., 2020; Lu et al., 2020; Wu et al., 2020; Zhou et al., 2020). Although viruses close to SARS-CoV-2 were discovered in pangolins (Lam et al., 2020; Xiao K. et al., 2020; Zhang et al., 2020), determining the intermediate host between bats and humans remains elusive (Boni et al., 2020; Han, 2020; Li et al., 2020; Liu P. et al., 2020). The secondary attack rate and therefore reproduction number of SARS-CoV-2 in humans are found to be much higher than those of SARS-CoV and MERS-CoV (Jing et al., 2020; Kucharski et al., 2020; Liu Y. et al., 2020; Madewell et al., 2020). Even transmission via aerosol of SARS-CoV-2 is possible (Morawska and Milton, 2020; Van Doremalen et al., 2020). The case-fatality is relatively low for SARS-CoV-2 (∼1%) compared with those of SARS-CoV (∼15%) and MERS-CoV (∼30%) (Mahase, 2020a; Peeri et al., 2020). These characteristics of SARS-CoV-2 appear to be typical features of human viruses (Figure 2).

Unfortunately, it seems unlikely that SARS-CoV-2 will disappear naturally or by current intervention strategies, including isolation of patients, quarantine of contacts, physical distancing, and standard precautions including wearing a mask and hand washing. It is possible to contain the outbreak at local level; some countries or regions were able to reduce their new cases to almost zero by extensive measures such as “lockdown” and strict “border control” (Baker M. et al., 2020; Erkhembayar et al., 2020; Wang C. J. et al., 2020; Wu and McGoogan, 2020). However, these interventions cannot be sustained for months or years worldwide (Chowdhury et al., 2020; Weill et al., 2020). Existence of asymptomatic and presymptomatic transmission makes public health intervention difficult (Fraser et al., 2004). Given high transmissibility (Kucharski et al., 2020; Liu Y. et al., 2020) and a high proportion of asymptomatic infections (Day, 2020; Oran and Topol, 2020), a subsequent wave of infections seems inevitable (Baker R. E. et al., 2020; Ferguson et al., 2020; Kissler et al., 2020; López and Rodó, 2020). As we discussed, a virus should avoid consumption of a susceptible population for sustained transmission. Ironically, mitigation strategies (i.e., “flattening the curve”) can ensure a susceptible uninfected population for a longer time, perhaps years (Kissler et al., 2020; Stewart et al., 2020). Seroprevalence against the virus is still low even in heavily affected areas by July 2020 (Eckerle and Meyer, 2020; Havers et al., 2020; Pollán et al., 2020; Stringhini et al., 2020). Further, regardless of mitigation, the virus can sustain transmission when immunity against it does not last long or virus keeps evolving to evade host immunity. Short immunity periods are the case for seasonal human coronaviruses (Callow et al., 1990; Galanti and Shaman, 2020; Kissler et al., 2020). Although natural infection with SARS-CoV-2 can induce neutralizing antibodies in humans (Brouwer et al., 2020; Chi et al., 2020; Ju et al., 2020; Shi R. et al., 2020), we still do not know if the infection confers immunity to prevent reinfection and, if so, for what duration (Altmann and Boyton, 2020; Ibarrondo et al., 2020; Isho et al., 2020; Kirkcaldy et al., 2020; Long et al., 2020; Robbiani et al., 2020; Staines et al., 2020). Many cases that tested positive after once turning negative are currently considered as reactivation, long-term shedding, or detection of residual genetic material (An et al., 2020; Mahase, 2020b; Xiao A. T. et al., 2020) although a few cases of possible reinfection have been reported (Bloomberg, 2020). There is also concern that preexisting immunity might worsen the disease severity by mechanisms called antibody-dependent enhancement (Arvin et al., 2020; Eroshenko et al., 2020).

Still, the virus could go extinct. A key for containment lies in how we reduce the pool of susceptible people. Reduction can be achieved by natural infection of most people and/or by the introduction of world-scale mass vaccination. We pointed out previously in this article that four factors are important for such eradication: (1) no animal reservoir, (2) a long-lasting immunity, (3) an adequate proportion of immunized people, and (4) a small proportion of asymptomatic infections (Figure 2). For SARS-CoV-2, (1) no known non-human animal reservoir exists, but the virus can infect other animals suggesting possible animal-to-human transmission (Newman et al., 2020; Oreshkova et al., 2020; Shi J. et al., 2020; Sit et al., 2020). (2) The titer of antibody against SARS-CoV-2 in patients has been observed to decline during the early convalescent phase (Ibarrondo et al., 2020; Long et al., 2020). Still, vaccine candidates for SARS-CoV-2 showed the efficacy to induce neutralizing antibodies in humans (Folegatti et al., 2020; Jackson et al., 2020; Mulligan et al., 2020; Zhu et al., 2020) and it is possible that not only humoral immunity but cellular immunity counteracts SARS-CoV-2 infections (Altmann and Boyton, 2020; Grifoni et al., 2020; Le Bert et al., 2020; Ni et al., 2020; Weiskopf et al., 2020). Protective immunity by infection or vaccination in animal models lasted for at least a short period (Chandrashekar et al., 2020; Corbett et al., 2020; Deng et al., 2020; Gao et al., 2020; van Doremalen et al., 2020; Wang H. et al., 2020; Yu et al., 2020). Further, cross-reactivity of immune response between SARS-CoV-2 and seasonal human coronaviruses has been reported; however, it is uncertain whether viral interference occurs between SARS-CoV-2 and other viruses (Baker R. E. et al., 2020; Braun et al., 2020; Grifoni et al., 2020; Kissler et al., 2020; Le Bert et al., 2020; Mateus et al., 2020; Ng et al., 2020; Sette and Crotty, 2020; Wec et al., 2020; Weiskopf et al., 2020). (3) We must produce large quantities of vaccines and distribute them even to remote areas and conflict zones. We are now struggling to meet this challenge for measles and polio eradication (Peck et al., 2019; Chard et al., 2020). Meanwhile, a recent analysis that considered the heterogeneity in infectivity among individuals showed herd immunity required to contain this outbreak could be considerably smaller than previously thought (Britton et al., 2020; Gomes et al., 2020). (4) Unfortunately, a substantial proportion of infections with the virus do not cause notable symptoms (Day, 2020; Oran and Topol, 2020), and asymptomatic/presymptomatic people can transmit infection (Bai et al., 2020; Furukawa et al., 2020; Furuse et al., 2020; He et al., 2020). Assessing outbreak containment and determining when and how the virus dies out are thus difficult.

## Concluding Remarks

Zoonotic viruses and human viruses have different characteristics, even though many human viruses originally come from animal viruses. The animal-human interface brought SARS-CoV-2 into human populations, and the virus has already acquired the ability for effective transmission among humans. Projecting the future of the current outbreak remains to be difficult. The virus could become a “human virus” and coexist in the world population. For this scenario, we can still hope that its morbidity and mortality lower as they do for post-pandemic influenza. Even if natural infection or vaccination cannot provide a long-lasting immunity to prevent reinfection, it could reduce the severity of the disease (Gao et al., 2020; Ng et al., 2020; Wang H. et al., 2020; Yu et al., 2020). Future development of antiviral agents could contribute to that as well (European Centre for Disease Prevention and Control, 2020; Siemieniuk et al., 2020; Wiersinga et al., 2020). It is less likely but still possible that the virus will go extinct. However, many obstacles exist for the latter scenario to materialize.

## Data Availability Statement

The original contributions presented in the study are included in the article/supplementary material, further inquiries can be directed to the corresponding author/s.

## Author Contributions

HO conceived the study. YF reviewed the literatures and wrote the first draft of the manuscript. Both authors reviewed the draft and approved the final version of the manuscript.

## Conflict of Interest

The authors declare that the research was conducted in the absence of any commercial or financial relationships that could be construed as a potential conflict of interest.
